# Study on Pharmacokinetic and Bioavailability of Tamarixetin after Intravenous and Oral Administration to Rats

**DOI:** 10.1155/2019/6932053

**Published:** 2019-12-10

**Authors:** Jiayuan Shen, Qi Jia, Xuhua Huang, Guangzhe Yao, Wenjuan Ma, Yanxu Chang, Huizi Ouyang, Jun He

**Affiliations:** ^1^First Teaching Hospital of Tianjin University of Traditional Chinese Medicine, Tianjin 300193, China; ^2^Tianjin State Key Laboratory of Modern Chinese Medicine, Tianjin University of Traditional Chinese Medicine, Tianjin 301617, China

## Abstract

In this study, a sensitive and reliable HPLC-MS/MS method was established to quantify tamarixetin in rat plasma. This method was then applied to research on the pharmacokinetic and bioavailability of tamarixetin after intravenous and oral administration *in vivo*. The study was performed on CORTECS C_18_ column (4.6 mm × 150 mm, 2.7 *μ*m) using mobile phase composed of methanol-water-formic acid (85 : 15 : 0.1, v/v) at a flow rate of 0.3 mL/min by a tandem mass system with an electrospray ionization (ESI) source in the negative multiple-reaction monitoring (MRM) mode. The calibration curves showed good linearity in the range of 5–4000 ng/mL. The intra- and interday precision of tamarixetin was less than 8.7% and 4.8%, respectively, and accuracy was within ±9.5%. Extraction recovery (91.4–100.0%) and matrix effect (99.4–107.4%) met the guidelines published by regulatory authorities. The oral bioavailability of tamarixetin was 20.3 ± 12.4%.

## 1. Introduction

Tamarixetin, the nickname of 4'-O-methyl quercetin, is a kind of flavonol group. The 4'-hydroxy position of the element has an O-methylation, and it is an O-methylated flavonol metabolized from quercetin [1, 2]. Gupta and Seshadri first reported tamarixetin in 1954, who isolated this ingredient from *Tamarix troupii* (a leaf of an ornamental plant) [3]. About 20 years ago, it was isolated and identified from *Astragalus Miser* var. oblongifolius, a plant named “poisonous vetch,” by Norris and Stermitz [4]. Today, tamarixetin can be obtained from plants or synthesized chemically, through selective methylation of 7-O-benzylquercetin tetraacetate and selective protection/deprotection of 3',4'-catechol of quercetin [1, 5].

As a flavonoid, tamarixetin has various pharmacological activities, such as being cardioprotective, gastroprotective, and having anti-inflammatory and antitumor effects [6–9]. It may play a role in enhancing the stability of hepatic metabolites and promoting intestinal absorption of flavonoids with less thiol toxicity [10, 11]. In addition, researchers have confirmed that tamarixetin has the effect of inhibiting liver cancer in PLC/PRF/5 and HepG2 cells [2]. Tamarixetin has better anti-inflammatory properties compared with quercetin during bacterial sepsis via increased interleukin (IL)-10 production [8]. This property is similar to previous research that PNIPAM nanoparticles of curcuminoids could significantly decrease levels of TNF-*α* and IL-1*β* [12]. Although tamarixetin has many pharmacological activities, its analytical studies are limited. Quercetin and its metabolites such as tamarixetin in rat plasma and urine were determined by UPLC for exploring its physicochemical properties, for instance small phenolic acids and derivatives with preserved flavonoid structure [13]. In addition, researchers have studied the pharmacokinetics of tamarixetin in Xiheliu extract by HPLC [14]; however, the detection time was 40 min, and the lower limit of quantification (LLOQ) was relatively high (1.6 *μ*g/mL), which might affect the accuracy of pharmacokinetic results. Therefore, a rapid and sensitive analysis method needs to be established for understanding the character and metabolism of tamarixetin itself *in vivo*.

In our study, an HPLC-MS/MS method was established and validated to study the pharmacokinetic rule and oral bioavailability of tamarixetin. This is the first study on bioavailability of tamarixetin following its oral and intravenous administration in rats. These data will provide theoretical basis for the metabolism of tamarixetin via oral and intravenous administration *in vivo*.

## 2. Materials and Methods

### 2.1. Chemicals and Reagents

Methanol and acetonitrile (HPLC grade) were obtained from Merck Co., Ltd. Formic acid (chromatographic purity) was purchased from ROE Co., Ltd. Ultrapure water was prepared by the Milli-Q water purification system (Millipore, Milford, MA, USA). Tamarixetin (purity ≥98.0%) and naringenin (internal standard, IS, purity ≥98.0%) were purchased from Chengdu Must Bio-Technology Co., Ltd. (Chengdu, China).

### 2.2. Instruments and Experimental Conditions

HPLC-MS/MS analysis was operated using an Agilent 1200 high-performance liquid chromatography coupled with an Aglient 6430 series triple quadrupole mass spectrometer with an electrospray ionization (ESI) source. CORTECS C_18_ column (4.6 mm × 150 mm, 2.7 *μ*m) was employed for the separation of tamarixetin and IS. The mobile phase components were methanol-water-formic acid (85 : 15 : 0.1, v/v), injected at a flow rate of 0.3 mL/min for isocratic elution. The column temperature and injection volume were optimized at 30°C and 5 *μ*L, respectively. All mass spectrometry data were acquired with Mass Hunter workstation software.

**Figure 1:**
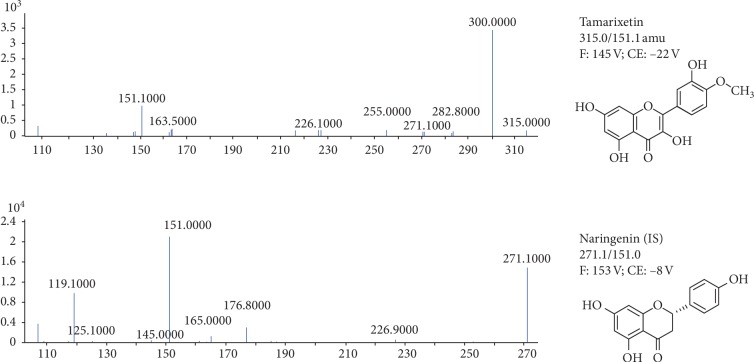
Chemical structures and mass spectrum of tamarixetin and IS.

The ESI source was performed in the negative ion mode and finalized as follows: capillary voltage was set at −4000 V for the negative ionization mode; drying gas temperature was 300°C, flow was 11 L/min, and nebulizing gas pressure was 15 psi. The optimal MRM fragmentation transitions were *m*/*z* 315.0 ⟶ 151.1 with a fragmentor voltage of 145 V and a collision energy (CE) of 22 V for tamarixetin and *m*/*z* 271.1 ⟶ 151.0 with a fragmentor voltage of 153 V and CE of 8 V, for naringenin (IS). The chemical structures and mass spectrum of tamarixetin and naringenin are shown in Figure 1.

### 2.3. Preparation of Calibration Standards, Quality Control Samples, and IS Solution

The stock solutions of tamarixetin and IS were weighed accurately, dissolved in methanol at a final concentration of 1 mg/mL, and stored at 4°C. A series of standard working solutions with tamarixetin at different concentrations were acquired by further dilution with methanol. The rat blank plasma was spiked with 20 *μ*L of working solutions and 20 *μ*L of IS for the preparation of calibration standards. The final concentrations of the analyte in rat plasma were over the range of 5–4000 ng/mL.

Quality control (QC) samples were prepared at three concentrations including low, medium, and high concentrations by similar procedure as the method above for calibration standards preparation.

### 2.4. Preparation of the Plasma Sample

100 *μ*L rat plasma was placed in a tube with IS (1 *μ*g/mL, 20 *μ*L) and methanol (20 *μ*L). After vortexing for 3 min, liquid-liquid extraction (LLE) was carried out using 3 mL of ethyl acetate. The mixture was then vortex-mixed for 3 min and centrifuged at 14,000 rpm for 10 min. The upper organic phase was collected to another clean tube and evaporated to dryness under a gentle stream of nitrogen. The dried residue was reconstituted in 100 *μ*L methyl alcohol and centrifuged at 14,000 rpm for 10 min [15]. Finally, 5 *μ*L of the supernatant was injected into the system for analysis.

### 2.5. Method Validation

#### 2.5.1. Specificity

The specificity was determined by blank plasma samples from six different rats with different concentrations of tamarixetin to evaluate whether the established extraction program and analysis method generated endogenous interference.

#### 2.5.2. Linearity and LLOQ

The linearity was evaluated by blank rat plasma with eight levels of the tamarixetin solution covering the range 5–4000 ng/mL and IS in duplicate over 3 consecutive days. The calibration curve was calculated by plotting peak area ratios of tamarixetin to the IS (*y*) versus the concentrations of the calibration standards (*x*), with 1/*x* as the weight factor. The LLOQ was determined at the lowest analytical concentration, with signal-to-noise ratio (S : N) at about 10.

#### 2.5.3. Precision and Accuracy

QC samples were analyzed at three concentration levels (5, 500, and 4000 ng/mL) in order to evaluate precision and accuracy. All concentrations were analyzed and replicated six times over three days for validation. Intra- and interday precision were evaluated by RSD, while accuracy was measured by RE.

#### 2.5.4. Extraction Recovery and Matrix Effect

The extraction recovery of analytes was measured by the peak area ratio of the prespiked standard solutions to the postspiked standard solutions. The matrix effect of tamarixetin was assessed by the ratio of the peak area of postspiked standard solutions to that of the standard tamarixetin solution. Both the extraction recovery and matrix effect were tested with three concentrations (5, 500, and 4000 ng/mL) in six parallels.

#### 2.5.5. Stability

The stability of tamarixetin in plasma samples was investigated by analyzing samples at three different concentration levels under different sets of conditions: stored at an autosampler for 6 h, at room temperature for 2 h, and under three freeze-thaw cycles. All stability tests were repeated six times in parallel.

### 2.6. Pharmacokinetic Study

Male Sprague-Dawley rats (230 ± 10 g) were purchased from Beijing HFK Experimental Anima Technology Co., Ltd. The animals were kept in standard conditions with alternating 12 h light and 12 h dark cycle at least 7 days before experiment. Before administration, rats were fasted for 12 h with free access to water. In order to perform bioavailability and pharmacokinetics of tamarixetin, rats were divided into two groups randomly (intravenous group and gavage group). Intravenous group was given tamarixetin at an oral dose of 2 mg/kg and gavage group at an oral dose of 20 mg/kg. The blood samples (200 *μ*L) were collected from the rat fossa orbitalis vein at 0, 0.03, 0.08, 0.17, 0.25, 0.5, 0.75, 1, 1.25, 1.5, 2, 4, 6, 8, 10, 12, and 24 h after oral administration. Blood samples were also collected at 0.03, 0.08, 0.17, 0.25, 0.33, 0.5, 0.75, 1, 2, 4, 6, 8, 10, 12, and 24 h after tail vein injection. All the plasma samples were centrifuged at 6,000 rpm for 10 min immediately. The supernatant was collected into clean centrifuge tubes, and the samples were processed according to the method of plasma sample preparation mentioned above. The processed samples were then analyzed by HPLC-MS/MS. Animal studies were approved and conducted in accordance with the Guidelines of Laboratory Animal Ethics Committee of Tianjin University Traditional Chinese Medicine (TCM-LAEC20180051).

### 2.7. Data Analysis

All pharmacokinetic parameters were computed by “Drug and Statistics 3.0” (DAS 3.0) (Medical College of Wannan, China). The absolute bioavailability (*F*) was calculated by the following equation:

(1) $$\begin{array}{c} F=\frac{\text{AUC}\left(\text{oral}\right)\times \text{dose}\left(\text{intravenous}\right)}{\text{AUC}\left(\text{intravenous}\right)\times \text{dose}\left(\text{oral}\right)}\times 100\%. \end{array}$$

## 3. Results

### 3.1. Optimization of HPLC-MS/MS Setting

This study aims to analyze tamarixetin and IS; hence, rapid analysis time and sensitive detection method were our study objectives. Different columns and mobile phases were tested, such as XBridge™ BEH C_18_ column (4.6 mm × 50 mm, 2.5 *μ*m), XBridge™ C_18_ column (2.1 mm × 100 mm, 3.5 *μ*m), CORTECS C_18_ column (4.6 mm × 150 mm, 2.7 *μ*m), methanol-water-formic acid, and acetonitrile-water-formic acid. For rapid, sensitive and efficient analytical method, CORTECS C_18_ column (4.6 mm × 150 mm, 2.7 *μ*m) and methanol-water-formic acid (85 : 15 : 0.1, v/v) mobile phase were chosen for the following analysis studies.

### 3.2. Optimization of Sample Preparation

During the whole experiment, sample handling plays an important role. We chose different sample processing methods for analysis, including liquid-liquid extraction (LLE) and protein precipitation (PPT). The results showed that the method of LLE with ethyl acetate met with satisfactory extraction recovery and matrix effect.

### 3.3. Method Validation

#### 3.3.1. Specificity

**Figure 2:**
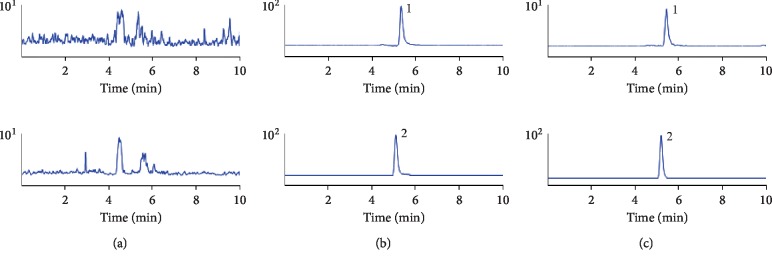
MRM chromatograms of tamarixetin and IS. Tamarixetin (1) and IS (2): (a) blank plasma; (b) blank plasma spiked with tamarixetin and IS; (c) plasma sample.

The representative chromatograms of tamarixetin and naringenin are displayed in Figure 2. The specificity was determined by comparing the chromatograms of blank plasma from six different rats with plasma samples containing tamarixetin. The retention time of tamarixetin and IS was 5.35 min and 5.11 min, respectively. There were no interferences observed in the MRM chromatograms of selected blank plasma, which suggested that blank plasma had no detectable interference when spiked with tamarixetin and the IS.

#### 3.3.2. Linearity and Sensitivity

As shown in Table 1, the calibration standard curve was obtained as *Y* = 0.1824*X* + 0.0026, which showed good linearity (*r*^2^ = 0.9982) within the scope of 5–4000 ng/mL for tamarixetin. In the above equation, the peak area ratios of tamarixetin to IS were defined as *Y*, and the plasma concentrations of tamarixetin were defined as *X*. In this range, the plasma concentration of target compound could be calculated accurately. The LLOQ was 5 ng/mL, which indicated that this method was sensitive, allowing for more accurate mean plasma concentration-time curves of tamarixetin.

#### 3.3.3. Precision and Accuracy

In this experiment, the precision was assessed by the relative standard deviation (RSD), and accuracy was expressed as relative error (RE) at three concentration levels in six replicates. The RSDs of the intra- and interday precision day were below 8.7%, and the RE of accuracy was within ±9.5%. Results displayed in Table 2 suggested that this method is accurate and has good repeatability.

#### 3.3.4. Extraction Recovery and Matrix Effect

The data of extraction recovery and matrix effect obtained are presented in Table 3. The extraction recovery and matrix effect of tamarixetin at three concentration levels were in the scope of 91.4%–100.0% and 99.4%–107.4%, respectively. These data indicated that the process of the study was efficient and reproducible. Meanwhile, the acceptable matrix effect suggested that there was no significant ion suppression or enhancement effects in all biological matrices.

#### 3.3.5. Stability

The stability test was demonstrated with tamarixetin in plasma under different storage conditions such as being stored at an autosampler for 6 h after preparation, at room temperature for 2 h, and at three freeze-thaw cycles. All data were summarized as in Table 4. The results indicated that tamarixetin had no significant degradation in rat plasma under the above storage conditions, and that the data of following experiments are reliable.

### 3.4. Pharmacokinetic Studies

**Figure 3:**
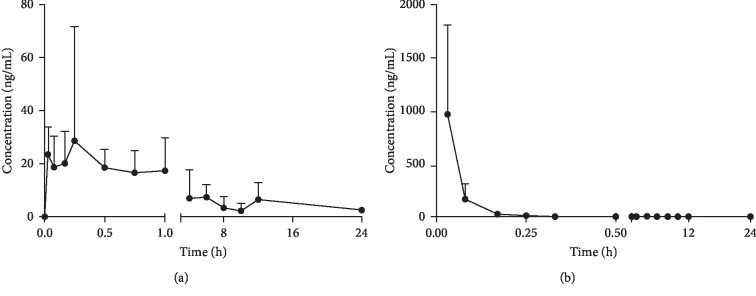
Mean plasma concentration-time curves of tamarixetin after oral (a) and intravenous (b) administration (mean ± SD, *n* = 6).

The above HPLC-MS/MS method successfully assessed the pharmacokinetics of orally (20 mg/kg) and intravenously (2 mg/kg) administered SD rats. The pharmacokinetic parameter data obtained for one-compartment modelling and mean plasma concentration-time profile for tamarixetin are shown in Table 5 and Figure 3, respectively.

#### 3.4.1. Pharmacokinetics of Tamarixetin in Rats after Intravenous Administration

On the basis of DAS evaluation, the one-compartment model was most suitable for analyzing the pharmacokinetics of tamarixetin. From the results, we found that the concentration of tamarixetin in rat plasma reached its maximum almost immediately. *T*_1/2_ of tamarixetin was 0.03 h, suggesting that the elimination of tamarixetin was rapid. In addition, the parameters of AUC_(0–24 h)_, AUC_(0–∞)_, MRT_(0–24 h),_ and MRT_(0-∞)_ were 134.29 ± 74.53 ng/mL·h, 138.63 ± 75.43 ng/mL·h, 3.07 ± 2.54 h, and 4.21 ± 3.15 h, respectively.

#### 3.4.2. Pharmacokinetics of Tamarixetin in Rats after Oral Administration

As aforementioned, the one-compartment model was a prime model for the data assessment of tamarixetin *in vivo* after oral administration. As shown in Table 5 and Figure 3, *T*_max_ of tamarixetin was 0.61 h, which demonstrated fast absorption. The *T*_1/2_ was calculated as 9.68 h, which revealed that the elimination of tamarixetin was slow with the route of oral administration. Furthermore, *C*_max_ in plasma sample after oral administration was 49.72 ± 38.31 ng/mL. The most probable reason for this finding might be its poor solubility speculated from its physicochemical properties [16]. The value of AUC_(0–24 h)_, AUC_(0–∞)_, MRT_(0–24 h),_ and MRT_(0–∞)_ was 163.63 ± 72.29 ng/mL·h, 226.08 ± 122.53 ng/mL·h, 6.81 ± 3.10 h, and 15.42 ± 10.43 h, respectively.

#### 3.4.3. Bioavailability of Tamarixetin in Rats after Administration

According to the equation, the oral bioavailability of tamarixetin obtained was 20.3 ± 12.4%. The cause of this phenomenon might be due to its poor aqueous solubility and poor absorption [17].

## 4. Discussion

In this research, we established a reliable and sensitive HPLC-MS/MS method to examine pharmacokinetics of tamarixetin after intravenous and oral administration *in vivo*. Sample treatment was performed according to the method of Shen [15], which yielded satisfactory results.

In the pharmacokinetics study, we evaluated the stability of tamarixetin in different storage conditions. From the preliminary experiment, we concluded that tamarixetin dissolved in methanol was kept stable at room temperature for 12 h. Nevertheless, its stability in plasma sample was not longer than 3 h, and hence plasma sample collected after administration should be analyzed immediately. Such instability in plasma could be because tamarixetin reacted with the plasma or produced other substances.

In the early stage of the experiment, many internal standards such as liquiritigenin, naringenin, and rutin were tested. Results showed that naringenin had a structure and chemical nature similar to tamarixetin, but did not react with the latter nor affected its content detection. Hence, naringenin was chosen as the IS in this study.

Results have shown that *T*_1/2_ of tamarixetin after intravenous administration was only 0.03 h, indicating that the concentration of tamarixetin in plasma was rapidly decreased. It was clearly seen that the drug concentration in plasma rapidly reached its peak value with a mean *C*_max_ of 967.93 ± 899.16 ng/mL. Moreover, *T*_1/2_ of the gavage group was calculated as 9.68 h, revealing that the elimination of tamarixetin was slow with the route of oral administration. Meanwhile, *C*_max_ of tamarixetin was reached quickly in plasma after dosing, at 49.72 ± 38.31 ng/mL. The oral bioavailability was calculated to be 20.3 ± 12.4%. Results suggested that oral absorption of tamarixetin is not ideal, probably due to its poor solubility as speculated from its physicochemical properties. Another possible explanation could be the distribution of tamarixetin in tissues and cells prior to the systemic circulation. Thus, the concentrations of tamarixetin in tissues and cells will be monitored in the future for better understanding in this phenomenon. This study would be helpful for future studies on the route of administration for tamarixetin.

In recent years, researchers have enhanced the oral bioavailability of drugs with the help of composite or biological materials. For example, mucoadhesive nanoemulsion, biodegradable polymeric nanoparticles, PLGA-polymeric nanoparticles, and chitosan nanoparticles were often used to enhance the oral bioavailability of certain drugs or herbs [18–23]. Further studies in this aspect would be helpful towards the clinical application of tamarixetin.

## 5. Conclusion

In summary, the HPLC-MS/MS method was established for the quantification of tamarixetin. This method was successfully used to explore the pharmacokinetic and oral bioavailability studies of tamarixetin after oral and intravenous administration *in vivo*. The oral bioavailability of tamarixetin was 20.3 ± 12.4%, and this result would be helpful towards optimizing its route of administration and providing reference for its further development.

## Figures and Tables

**Table 1:** Calibration curves, correlation coefficients, linear ranges, and LLOQ of tamarixetin.

Compound	Calibration curves	Correlation coefficients (*r*^2^)	Linear range (ng/mL)	LLOQ (ng/mL)
Tamarixetin	*Y* = 0.1824*X* + 0.0026	0.9982	5–4000	5

**Table 2:** Precision and accuracy of tamarixetin in rat plasma (*n* = 6).

Compounds	Spiked concentration (ng/mL)	Intraday			Interday		
		Measured concentration (ng mL^−1^)	Accuracy (RE, %)	Precision (RSD, %)	Measured concentration (ng mL^−1^)	Accuracy (RE, %)	Precision (RSD, %)
Tamarixetin	5	4.6 ± 0.4	−8.0	8.7	4.8 ± 0.2	−4.0	4.2
	500	547.4 ± 18.1	9.5	3.3	512.7 ± 24.5	2.5	4.8
	4,000	3935.8 ± 119.4	−1.6	3.0	4034.4 ± 151.5	0.9	3.8

**Table 3:** . Extraction recoveries and matrix effects of the analytes (*n* = 6).

Compounds	Concentration (ng/mL)	Extraction recovery (%)	RSD (%)	Matrix effect (%)	RSD (%)
Tamarixetin	5	100.0 ± 7.1	7.1	99.4 ± 8.0	8.0
	500	92.1 ± 3.5	3.8	107.4 ± 5.4	5.0
	4000	91.4 ± 3.9	4.3	102.3 ± 3.2	3.1

**Table 4:** Stability of all analytes in rat plasma (*n* = 6).

Compounds	Spiked concentration (ng/mL)	Room temperature for 2 h		Three freeze-thaw		Autosampler for 6 h	
		Measured (ng/mL)	RSD (%)	Measured (ng/mL)	RSD (%)	Measured (ng/mL)	RSD (%)
Tamarixetin	5	4.7 ± 0.3	6.4	4.9 ± 0.3	6.1	4.7 ± 0.4	8.5
	500	524.3 ± 35.8	6.8	519.3 ± 27.6	5.3	478.3 ± 10.1	2.1
	4000	4005.7 ± 435.5	10.9	3989.8 ± 259.9	6.5	3687.5 ± 41.9	1.1

**Table 5:** Pharmacokinetic parameters of tamarixetin after intravenous and oral administration (*n* = 6).

Parameters	Intravenous (2 mg/kg)	Oral administration (20 mg/kg)
*T*_max_ (h)	0.03 ± 0.00	0.61 ± 0.36
*C*_max_ (ng/mL)	967.93 ± 899.16	49.72 ± 38.31
AUC_(0–24 h)_ (ng/mL·h)	134.29 ± 74.53	163.63 ± 72.29
AUC_(0–∞)_ (ng/mL·h)	138.63 ± 75.43	226.08 ± 122.53
*T*_1/2_ (h)	0.03 ± 0.01	9.68 ± 9.45
MRT_(0–24 h)_ (h)	3.07 ± 2.54	6.81 ± 3.10
MRT_(0–∞)_ (h)	4.21 ± 3.15	15.42 ± 10.43

## Data Availability

The data used to support the findings of this study are available from the corresponding author upon request.
